# High UBE2D1 expression is associated with poor prognosis and immunotherapy resistance in head and neck cancer

**DOI:** 10.1016/j.jobcr.2026.101423

**Published:** 2026-02-14

**Authors:** Prathibha Prasad, Paramasivam Arumugam

**Affiliations:** a Basic Medical and Dental Sciences Department, College of Dentistry, Ajman University, Ajman, United Arab Emirates; b Center for Medical and Bio-Allied Health Sciences Research, Ajman University, Ajman, United Arab Emirates; c Department of Oral Pathology, Saveetha Dental College and Hospital, Saveetha Institute of Medical and Technical Sciences (SIMATS), Saveetha University, Chennai, India; d Molecular Biology Lab, Saveetha Dental College and Hospital, Saveetha Institute of Medical and Technical Sciences (SIMATS), Saveetha University, Chennai, India

**Keywords:** HNSCC, Healthcare, UBE2D1, Ubiquitin, Prognostic marker

## Abstract

**Background:**

Head and neck squamous cell carcinoma (HNSCC) is an aggressive malignancy with poor clinical outcomes. Ubiquitin-conjugating enzyme E2 D1 (UBE2D1), a key component of the ubiquitination machinery, has been implicated in tumor progression in several cancers; however, its relevance in HNSCC remains unclear. This study aimed to investigate UBE2D1 expression in HNSCC, evaluate its prognostic significance while acknowledging disease heterogeneity, including HPV and TP53 status, and explore its potential association with response to anti-PD-1 immunotherapy.

**Methods:**

UBE2D1 mRNA and protein expression were analyzed using the Cancer Genome Atlas Head and Neck Squamous Cell Carcinoma (TCGA-HNSCC) dataset via the UALCAN platform. Transcript-level expression was validated by quantitative real-time PCR in paired HNSCC and adjacent normal tissues. Protein–protein interaction and functional enrichment analyses were performed to examine UBE2D1-associated molecular networks. Prognostic relevance was assessed using Kaplan–Meier survival analysis.

**Results:**

UBE2D1 mRNA and protein expression were significantly elevated in HNSCC compared with normal tissues (*P* < 0.05). Higher UBE2D1 expression was associated with poorer overall and relapse-free survival. Exploratory analyses suggested a weak association between elevated UBE2D1 expression and response patterns to anti-PD-1 therapy. Functional analyses identified associations with oncogenic pathways, with UBE2D1 expression positively correlated with MMP2 and negatively correlated with SMAD4, indicating a potential role in tumor progression.

**Conclusion:**

UBE2D1 is frequently overexpressed in HNSCC and is associated with adverse clinical outcomes. Although its association with immunotherapy response requires further validation, UBE2D1 may be a prognostic biomarker in HNSCC.

## 1 Introduction

Head and neck squamous cell carcinoma (HNSCC) encompasses a group of malignancies affecting the oral cavity, pharynx, hypopharynx, larynx, nasal cavity, and salivary glands, collectively ranking as the seventh most prevalent cancer globally. With 890,000 new cases and 450,000 deaths annually per GLOBOCAN estimates, HNSCC accounts for roughly 4.5% of cancer diagnoses and deaths 1, 2, 3. HNSCCs arise in the mucosal linings of the upper aerodigestive tract and are unexpectedly heterogeneous in nature. Classical risk factors include smoking and excessive alcohol consumption, and in recent years, the role of human papillomavirus (HPV) has emerged, particularly in oropharyngeal tumors 4, 5, 6.

Ubiquitination is a biological process in which the targeting proteins are modified with ubiquitin for degradation. DNA repair, proteasomal degradation, and cellular homeostasis all depends on this process. Enzymes belonging to at least three classes are involved in ubiquitination: ubiquitin-activating enzymes (E1s), ubiquitin-conjugating enzymes (E2s), and ubiquitin-protein ligases (E3s).^7^^,^^8^ Ubiquitination plays crucial roles in physiological processes and regulates both tumor-promoting and tumor-suppressing pathways.^9^ The ubiquitin-conjugating enzyme E2 D1 (UBE2D1) is a member of UBE2D family, which has been found to play a role in some important pathways of carcinogenesis.^10^

Previous studies reported that upregulated UBE2D1 is found in several cancers including non-small-cell lung cancer, osteosarcoma, and hepatocellular carcinoma 11, 12, 13. Emerging evidence links UBE2D1 to key tumor suppressor pathways frequently altered in HNSCC, including SMAD4-mediated TGF-β signaling and p53-associated ubiquitination. Given that SMAD4 loss and TP53 mutations are hallmark molecular events in HNSCC, these mechanistic associations provide a strong biological rationale for investigating UBE2D1 in HNSCC progression and therapeutic response.^14^ These mechanistic links suggest that UBE2D1 may play a biologically plausible role in HNSCC progression rather than representing a nonspecific ubiquitination marker. However, the role of UBE2D1 in HNSCC remains unclear. To fill this gap, our study aims to analyze the expression of UBE2D1 in HNSCC and examine its relationship with various clinicopathological features, prognosis, and response to immunotherapy. Additionally, we perform functional enrichment analyses to examine the protein network associated with UBE2D1.

## 2 Materials and methods

### 2.1 UBE2D1 expression analysis

UBE2D1 mRNA expression across multiple cancer types was initially explored using TIMER 2.0 (http://timer.comp-genomics.org), which provides comprehensive transcriptomic profiles derived from TCGA datasets. The University of Alabama at Birmingham (UALCAN) (http://ualcan.path.uab.edu) was used to analyze the TCGA dataset for a comprehensive pan-cancer analysis of UBE2D1 expression across 33 different cancer types. UBE2D1 expression levels across 30 different normal human tissues were examined using TPM values from the Human Protein Atlas (https://www.proteinatlas.org/).^15^ The current study primarily utilized the TCGA-HNSCC cohort for analysis, which comprises HNSCC tumor tissues (n = 520) and normal tissues (n = 44) from HNSCC patients. Proteomic data of HNSCC (n = 108) and matched normal tissues (n = 71) were obtained from the TCGA database. Differential expression analysis between tumor and normal tissues was performed. Protein expression of UBE2D1 was also assessed using immunohistochemistry (IHC) images and staining data from the Human Protein Atlas (HPA) database. No immunohistochemical analysis was performed on the authors’ in-house cohort. Clinical information for HNSCC patients from the TCGA dataset is presented in Supplementary Table 1.

### 2.2 Sample collection and UBE2D1 mRNA validation by qPCR

In this study, 26 paired HNSCC and normal tissue samples were collected during surgical resection at the Department of Oral Maxillofacial Surgery, Saveetha Dental College and Hospitals, Chennai. The samples were immediately stored at −80 °C. This study was approved (IEC No: SDC/FAC-12/19/003) and conducted in compliance with the ethical guidelines of the Institutional Ethics Committee and followed the principles outlined in the Declaration of Helsinki. Informed consent was obtained from all participants. HPV status was unavailable for all cases in the in-house cohort; therefore, qRT-PCR–based validation of UBE2D1 expression was performed without stratification by HPV status. The clinicopathological characteristics of the validation cohort, including tumor subsite and stage, are summarised in Supplementary Table 2.

TRIzol reagent (Thermo Fisher Scientific Inc., USA) was used to extract total RNA, and Nanodrop One was employed to measure the RNA's quantity and quality. Complementary DNA (cDNA) synthesis was performed using the Prime Script 1st Strand cDNA Synthesis Kit (Takara Bio Inc., Tokyo, Japan). The SYBR Green qPCR Kit was utilized to carry out qPCR reactions in a Bio-Rad CFX Opus qPCR. Primers used in this study for quantitative real-time PCR (qPCR) are listed in Table 1. Relative gene expression levels were calculated using the 2^−ΔΔCT^ method and normalized to glyceraldehyde 3-phosphate dehydrogenase (GAPDH).

**Table 1:** Primer sequence for qPCR.

Gene	Forward Primer	Reverse primer
*UBE2D1*	5′-AGCGCATATCAAGGTGGAG-3′	5′-AGAGCTGGTGACCATTGTG-3′
*GAPDH*	5′-TCCAAAATCAAGTGGGGCGA-3′	5′-TGATGACCCTTTTGGCTCCC-3′

### 2.3 Survival and immunotherapy response analysis

Prognostic relevance of UBE2D1 expression was analyzed through Kaplan-Meier Plotter (https://kmplot.com/analysis/),^15^ evaluating overall survival (OS) and relapse-free survival (RFS) in TCGA-derived HNSCC cohorts, as longitudinal survival data were not available for the in-house samples. UBE2D1 expression and its association with immunotherapy responses were analyzed using the ROC Plotter, based on publicly available GEO-derived HNSCC cohorts comprising patients treated with anti-PD-1 therapy, as integrated into the ROC Plotter database (https://rocplot.com/).

### 2.4 Functional enrichment analyses

Protein–protein interaction (PPI) networks were constructed using the STRING (https://string-db.org) database, and pathway/disease enrichment analyses were conducted to identify biological functions and clinical relevance. Gene Ontology (GO) and KEGG pathway enrichment analyses were conducted using Metascape (https://metascape.org) to explore the biological processes and signaling pathways associated with UBE2D1. Correlation analyses were performed between UBE2D1 expression and its targets, such as MMP2 and SMAD4, using both Pearson and Spearman correlation coefficients.

### 2.5 Statistical analysis

Statistical analyses were conducted using GraphPad Prism (GraphPad Software). Various statistical tests, including Analysis of Variance (ANOVA), Student's t-test, and Pearson's correlation, were used to compare groups. To evaluate patient survival, the Kaplan-Meier method was used, along with the log-rank test. Significance was set at *P* < 0.05. All data are expressed as the mean deviation from three independent experiments.

## 3 Results

### 3.1 UBE2D1 mRNA expression significantly increased in HNSCC

**Fig. 1:**
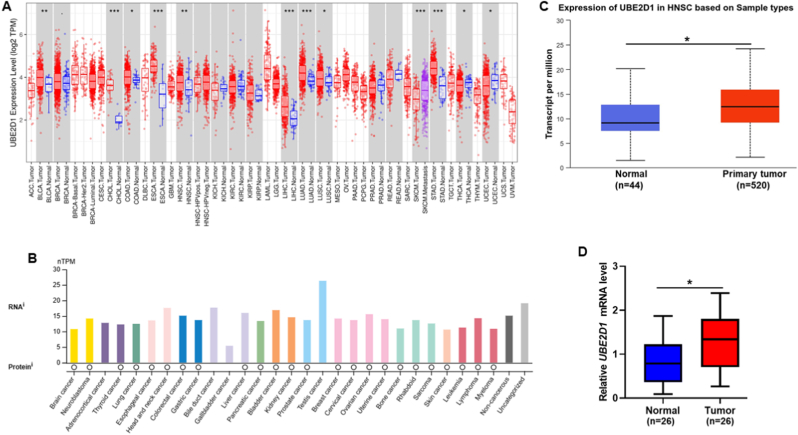
*UBE2D1* mRNA expression in HNSCC patients. (A) Comparison of *UBE2D1* mRNA expression between various tumor and non-tumor tissues. (B) *UBE2D1* mRNA expression in various cancer cells, including HNSCC. (C) Comparison of *UBE2D1* mRNA expression between HNSCC tumor (n = 520) and non-tumor tissues (n = 44). (D) Validation of *UBE2D1* mRNA expression in HNSCC (n = 26) and non-tumor tissues (n = 26) using qPCR. ∗*p <* 0.05, ∗∗*p <* 0.01, ∗∗∗*p <* 0.001.

In our study, we examined *UBE2D1* mRNA expression through a combination of data mining in online databases and experimental validation. Initial data analysis using TIMER2.0 and the Human Protein Atlas database revealed significant alterations in *UBE2D1* mRNA expression across various cancer tissues and cell lines (Fig. 1A and B, *P* < 0.05). UALCAN database analysis showed that *UBE2D1* mRNA expression significantly increased in HNSCC patient tissues (n = 520) compared to normal tissues (n = 44) (Fig. 1C, *P* < 0.05). Our validation study using qPCR confirmed the high expression of *UBE2D1* mRNA in tumor tissues (n = 26) compared with normal tissues (n = 26) (Fig. 1D, *P* < 0.05). The qPCR validation was conducted on paired HNSCC and adjacent normal tissues, without subgroup analysis by HPV status, due to incomplete HPV annotation in the in-house cohort.

### 3.2 UBE2D1 high expression associated with poor survival and immunotherapy resistance

**Fig. 2:**
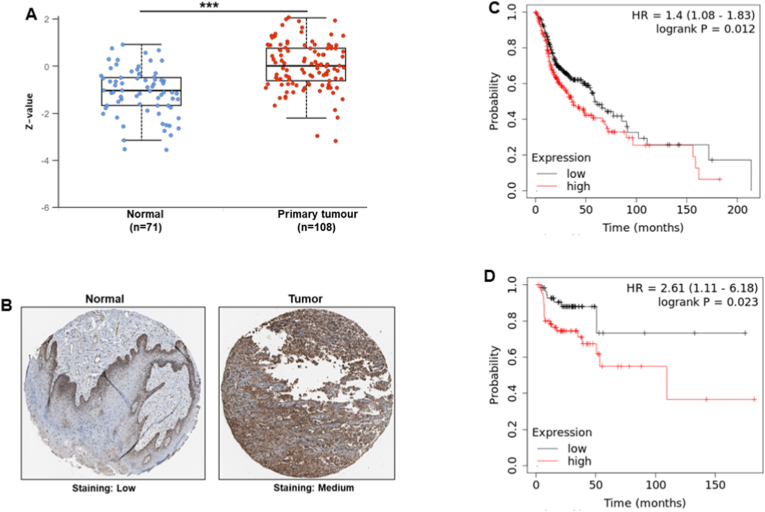
UBE2D1 protein expression and survival of HNSCC patients. (A) Comparison of UBE2D1 protein expression between HNSCC tumor and non-tumor tissues. (B) Representative immunohistochemistry images of UBE2D1 expression in HNSCC and normal tissues obtained from the Human Protein Atlas database. High expression of UBE2D1 protein in HNSCC tissue (Medium) compared with normal tissues (Low). High *UBE2D1* mRNA expression is associated with poor survival of HNSCC patients, including (C) overall survival, and (D) relapse-free survival. ∗∗∗*p <* 0.001.

In addition to high *UBE2D1* mRNA expression, the analysis of its protein expression exhibited consistent results. The UALCAN database analysis indicated that UBE2D1 protein levels were significantly upregulated in primary HNSCC tumors compared to adjacent non-tumor tissues (Fig. 2A, *P* < 0.001). Immunohistochemistry analysis further confirmed the higher UBE2D1 protein expression in HNSCC tumor tissues than normal tissues (Fig. 2B). Kaplan-Meier plotters demonstrated that patients with high UBE2D1 expression had a significantly shorter overall survival (Fig. 2C, HR = 1.4, *P* = 0.012) and relapse-free survival (Fig. 2D, HR = 2.61, *P* = 0.023).

**Fig. 3:**
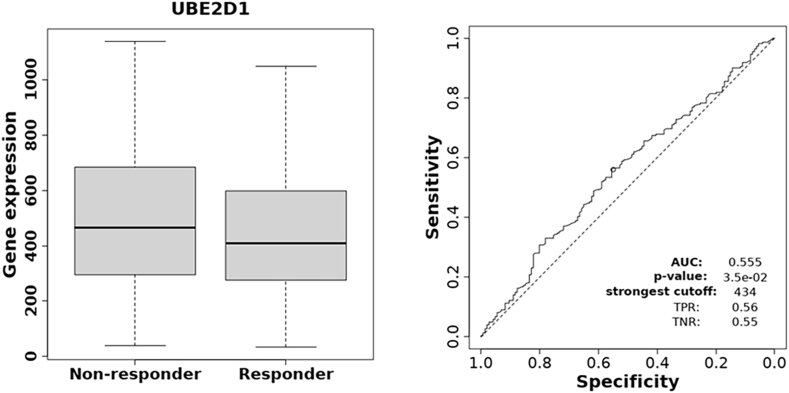
The association of *UBE2D1* expression and immunotherapy response. The receiver operating characteristic (ROC) curve analysis shows the predictive ability of UBE2D1 expression for response pattern to anti-PD-1 therapy (*P* = 3.5e.02). The ROC curve demonstrates weak discriminatory ability (AUC = 0.555) between responders and non-responders. The boxplot shows significantly higher UBE2D1 expression in non-responders than in responders to anti-PD-1 therapy.

To assess the predictive significance of UBE2D1 expression in immunotherapy response, we conducted ROC curve and boxplot analysis on anti-PD-1 treated cohorts (Fig. 3, *P* = 3.5e.02). ROC curve analysis demonstrated a weak discriminatory ability of UBE2D1 expression for differentiating responders from non-responders to anti-PD-1 therapy (AUC = 0.555). Consistent with this, UBE2D1 expression levels were modestly higher in non-responders compared with responders, suggesting a potential but limited association with immunotherapy response patterns (*P*-value: 3.5e-02). These results suggest that elevated UBE2D1 expression may serve as a predictive biomarker of resistance to anti-PD-1 immunotherapy. Together, these results suggest that UBE2D1 expression is upregulated in HNSCC and is negatively correlated with the prognosis and response to immunotherapy in HNSCC patients.

### 3.3 The carcinogenic role of UBE2D1 in HNSCC

**Fig. 4:**
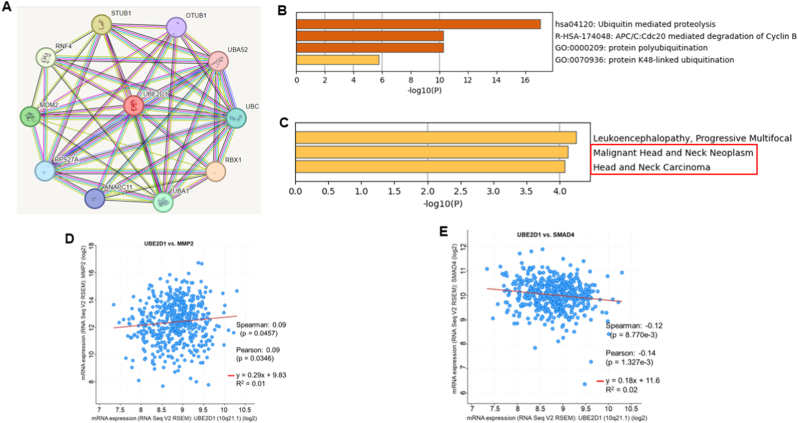
Functional pathway enrichment analysis. (A) Interacting networks of the UBE2D1 protein with other oncoproteins using the STRING databases. (B, C) Functional enrichment analysis of the UBE2D1 protein interaction networks associated with crucial pathways in HNSCC development and progression. UBE2D1 expression passively correlated with MMP2 expression (D) and negatively correlated with SMAD4 expression (E).

UBE2D1 interacts with UBC, MDM2, RBX1, and STUB1, as revealed by PPI network research analysis (Fig. 4A). Pathway enrichment study revealed that UBE2D1 involved in ubiquitin mediated proteolysis, protein polyubiquitination, and protein k48-linked ubiquitination (Fig. 4B). Disease enrichment analysis revealed significant associations with cancers including HNSCC development and progression (Fig. 4C). There is a significant positive correlation between UBE2D1 and MMP2 expression (Fig. 4D), which suggests that it may play a role in remodeling the extracellular matrix. Additionally, a negative correlation between SMAD4 and UBE2D1 expression (Fig. 4E) suggests that there may be a potential inhibition of tumor suppressor pathways.

## 4 Discussion

Our findings demonstrate that UBE2D1 is significantly upregulated in HNSCC, as confirmed by TCGA data and experimental validation. UBE2D1 protein expression was inferred from publicly available proteomic and immunohistochemical datasets and was not independently validated in the in-house cohort; thus, these findings should be interpreted as supportive public-dataset evidence rather than direct experimental confirmation. Elevated UBE2D1 expression is associated with poor overall and relapse-free survival. The survival rate among advanced HNSCC patients is only 34.9% and the effective multidisciplinary treatment for HNSCC is still limited.^16^^,^^17^ Thus, there is an urgent need for novel biomarkers and new therapeutic options for HNSCC.

Guan et al., reported that UBE2D1 regulates the development of various cancers. There was a negative correlation between its expression and overall survival in individuals with a number of malignancies, including breast cancer, stomach adenocarcinoma, liver hepatocellular carcinoma, and esophageal carcinoma.^18^ UBE2D1 was also found to be overexpressed in precancerous lesions, and was only upregulated in cancer cells rather than in normal liver cells, suggesting its potential as an early-stage hepatocellular carcinoma (HCC) marker.^13^

In colorectal cancer, UBE2D1 plays a role in the dysregulation of the Wnt and Ras-MAPK signaling pathways that are impacted by Aurora kinase A.^19^ While UBE2D1's silencing inhibited cell migration *in vitro* and *in vivo*. Its participation in malignancy and cancer growth was highlighted by Xie et al., who revealed that it was significantly elevated in gastric cancer (GC) and associated with poor OS.^20^ Research indicates that UBE2D1 DNA amplification, prevalent in lung adenocarcinoma (16.0%), is linked to increased RNA expression, and its upregulation is associated with unfavorable outcomes in both lung adenocarcinoma and bladder cancer.^11^^,^^21^

UBE2D1 knockdown inhibited migration in gastric cancer cells. Additionally, it decreased SMAD4 ubiquitination and MMP2/MMP9 levels. Low SMAD4 was associated with elevated MMP9, contributing to colorectal cancer malignancy.^22^ Silencing UBE2D1 has been reported to reduce SMAD4 ubiquitination and inhibit gastric cancer cell migration via the TGF-β/SMAD4 pathway. Persistence of the RNF128 Iso1–UBE2D1 complex stabilizes mutant p53 and promotes survival of Barrett's esophagus cells.^23^ Our study highlights that UBE2D1 is significantly associated with MMP2 and SMAD4 expression and plays a crucial role in HNSCC progression.

Although elevated UBE2D1 expression showed a weak association with response patterns to anti-PD-1 therapy, the limited predictive performance indicates that UBE2D1 alone is unlikely to serve as a robust biomarker of immunotherapy response. The higher levels of UBE2D1 in non-responders support the notion that dysregulated ubiquitin-conjugating enzymes may impact the effectiveness of immune checkpoints by modulating tumor immune evasion mechanisms. Therefore, UBE2D1 is frequently upregulated across cancers and linked to poor prognosis, tumor progression, and therapy resistance, highlighting its potential as a biomarker and therapeutic target. Future studies are needed to clarify the mechanistic role of UBE2D1 in immune resistance and to validate these findings in larger, independent cohorts. Additionally, combining UBE2D1 expression with other molecular biomarkers may further improve predictive accuracy in precision immunotherapy.

This study integrates large-scale public transcriptomic and proteomic datasets with independent qPCR validation in paired HNSCC tissues, enhancing the robustness and reproducibility of the findings. The use of complementary bioinformatics, survival, and exploratory immunotherapy response analyses further strengthens the consistency and clinical relevance of the results, particularly by focusing on a biologically plausible ubiquitination-related enzyme. Nevertheless, this study has certain limitations. The analyses rely partly on bioinformatics data with limited experimental validation. Incomplete HPV status and TP53 mutation data in the in-house cohort, together with a limited sample size and incomplete annotation in immunotherapy datasets, precluded robust subgroup analyses. Additionally, the absence of functional assays, such as knockdown or overexpression models, limits causal interpretation, and validation in samples from clinical immunotherapy responders and non-responders is warranted. These limitations highlight important directions for future investigation.

## 5 Conclusion

UBE2D1 is significantly upregulated in HNSCC at both the transcript and protein levels. Overexpression of UBE2D1 is associated with poor patient survival and resistance to immunotherapy. These findings suggest that UBE2D1 may serve as a potential predictive biomarker and warrant further functional and clinical validation.

## Patient consent

Not Applicable.

## Authors contribution

PP: Data curation, Formal analysis, Writing - original draft, review.

AP: Conceptualization, methodology, formal analysis, writing, reviewing, and editing. All authors reviewed the results and approved the final version of the manuscript.

## Ethical clearance

Ethical clearance for the study was obtained.

Institutional Human Ethical Committee (IHEC) with reference number (IEC No: SDC/FAC-12/19/003)

## Funding

The authors received no support from any organization for the submitted work.

## Declaration of competing interest

The authors declare that they have no known competing financial interests or personal relationships that could have appeared to influence the work reported in this paper.
