# Supplementary material for: High UBE2D1 expression is associated with poor prognosis and immunotherapy resistance in head and neck cancer
Source: J Oral Biol Craniofac Res. 2026 Feb 14;16(2):101423. doi: 10.1016/j.jobcr.2026.101423 (PMC12925490; doi:10.1016/j.jobcr.2026.101423)
Supplement: Multimedia component 1 [file mmc1.pdf]

Table S1: The clinical characteristics of patients with HNSCC from the TCGA-HNSCC dataset

S.No.	Variable	Category	No. of patients
1	Total samples	Primary tumor samples	520
2	Sample type	Tumor tissue	520
		Normal tissue	44
3	Age group	21–40 years	20
		41–60 years	236
		61–80 years	237
		8 –100 years	24
4	Gender	Male	383
		Female	136
5	Race	Caucasian	444
		African-American	47
		Asian	11
6	Tumor grade	Grade 1 (Well differentiated)	62
		Grade 2 (Moderately differentiated)	303
		Grade 3 (Poorly differentiated)	125
		Grade 4 / Unknown	7
7	Cancer stage	Stage I	27
		Stage II	71
		Stage III	81
		Stage IV	264
8	Lymph node metastasis	N0	176
		N1	67
		N2	12
		N3	8
9	HPV status	HPV-Positive	80
		HPV-Negative	434
10	TP53 mutation status	Mutant	327
		Wild-type	175
