# Supplementary material for: High UBE2D1 expression is associated with poor prognosis and immunotherapy resistance in head and neck cancer
Source: J Oral Biol Craniofac Res. 2026 Feb 14;16(2):101423. doi: 10.1016/j.jobcr.2026.101423 (PMC12925490; doi:10.1016/j.jobcr.2026.101423)
Supplement: Multimedia component 2 [file mmc2.docx]

**Table S2: The clinicopathological characteristics of the validation cohort**

| **S.No.** | **Variable** | **Category** | **No. of patients (%)** |
| --- | --- | --- | --- |
| 1 | Gender | Male | 21 (80.8) |
|  |  | Female | 5 (19.2) |
| 2 | Age | < 50 years | 11 (42.3) |
|  |  | ≥ 51 years | 15 (57.7) |
| 3 | Grade | Well differentiated | 15 (57.7) |
|  |  | Moderately differentiated | 9 (34.6) |
|  |  | Poorly differentiated | 2 (7.7) |
| 4 | Site | Buccal | 8 (30.8) |
|  |  | Tongue | 6 (23.1) |
|  |  | Other (RMT, GBS, Maxilla, Mandible) | 12 (46.1) |
| 5 | Stage | I | 4 (15.4) |
|  |  | II | 6 (23.1) |
|  |  | III | 5 (19.2) |
|  |  | IV | 11 (42.3) |
| 6 | Laterality | Left | 9 (34.6) |
|  |  | Right | 17 (65.4) |
| 7 | Lymph node metastasis | Yes | 11 (42.3) |
|  |  | No | 15 (57.7) |
